# Takayasu arteritis: clinical importance of extra-vessel uptake on FDG PET/CT

**DOI:** 10.1186/s41824-019-0059-1

**Published:** 2019-07-26

**Authors:** Junichi Tsuchiya, Daisuke Tezuka, Yasuhiro Maejima, Hyeyeol Bae, Takumi Oshima, Tomohiro Yoneyama, Kenzo Hirao, Mitsuaki Isobe, Ukihide Tateishi

**Affiliations:** 1 Department of Diagnostic Radiology and Nuclear Medicine, Tokyo Medical and Dental University, 1-5-45 Yushima, Bunkyo-ku, Tokyo, 113-8510 Japan; 2 Department of Cardiovascular Medicine, Tokyo Medical and Dental University, Tokyo, Japan; 3 Sakakibara Memorial Hospital, Tokyo, Japan

**Keywords:** Takayasu arteritis, [F-18]FDG PET/CT, Extra-vessel uptake

## Abstract

**Background:**

[F-18]fluorodeoxyglucose positron emission tomography/computed tomography is routinely used for assessing Takayasu arteritis patients. However, extra-vessel [F^-18^]fluorodeoxyglucose uptake has not been evaluated in detail in these patients. We aimed to describe the extent and distribution of extra-vascular [F-18]fluorodeoxyglucose uptake on positron emission tomography/computed tomography in Takayasu arteritis patients. Seventy-three [F-18]fluorodeoxyglucose positron emission tomography/computed tomography scans from 64 consecutive Takayasu arteritis patients (59 women, mean age, 35.4 years; range, 13 to 71 years) and 40 scans from age-matched controls (36 women, mean age, 37.8 years; range, 13 to 70 years) were examined. We graded [F-18]fluorodeoxyglucose uptake in large vessels using a 4-point scale and evaluated extra-vessel findings. Factors correlated with disease activity were examined. We evaluated the relationship between disease activity according to the National Institutes of Health score with extra-vessel findings, as well as other inflammatory markers (e.g., white blood cell count and C-reactive protein level).

**Results:**

Extra-vessel involvement was present in 50 of 73 (68.4%) scans, specifically at the following sites: lymph nodes, 1.4%; thyroid glands, 17.8%; thymus, 11.0%; spleen, 1.4%; vertebrae, 45.2%; and pelvic bones, 9.6%. Takayasu arteritis patients had higher [F-18]fluorodeoxyglucose uptake in the spine (*P* = 0.03) and thyroid glands (*P* = 0.003) than did controls; uptake in other regions was comparable between groups. Compared with inactive patients, those with active Takayasu arteritis had a higher number of [F-18]fluorodeoxyglucose uptake sites in the vasculature (*P* = 0.001). Finally, patients with a National Institutes of Health score of ≥ 1 had significantly higher extra-vascular involvement (*P* = 0.008).

**Conclusions:**

Extra-vessel [F-18]fluorodeoxyglucose uptake may be present in the context of Takayasu arteritis-related inflammatory processes. Information on extra-vascular [F-18]fluorodeoxyglucose uptake may be useful for detecting and evaluating inflammatory processes when interpreting positron emission tomography/computed tomography scans obtained from Takayasu arteritis patients.

## Background

Large vessel vasculitis, which includes Takayasu arteritis (TA) and giant cell arteritis, is a rare, chronic inflammatory disease that affects the walls of the aorta and its main branches, as well as the coronary and pulmonary arteries. The incidence of TA is estimated to be two in 1 million individuals; this disease has a high prevalence in Japan (Toshihiko 1996). The mean age of onset is 35 years, and women are disproportionately affected by this disease (2 to 25-fold higher). TA is potentially life-threatening, with a mortality rate as high as 35%. Although autoimmune processes driven by antigens have been speculated to cause TA, no specific antigen or stimulus has been identified, and its pathogenesis remains unknown (Kerr et al. 1994; Blockmans et al. 2009).

TA involves non-specific symptoms, such as fever and general weakness. Hence, diagnosis and evaluation of disease activity are difficult. Parameters such as erythrocyte sedimentation rate (ESR) and C-reactive protein (CRP) levels are also non-specific and contribute little towards the estimation of disease activity (Hoffman and Ahmed 1998).

Considering that the management of TA involves long-term treatment with steroids, it is imperative to make an accurate diagnosis. Diagnoses are typically made using multiple modalities, including ultrasound (US), angiography, computed tomography (CT), and magnetic resonance imaging (MRI). Angiography has been used to evaluate vessel stenosis, occlusion, and aneurysm formation. However, angiography-based diagnoses can be operator-dependent and subjective. Aortic wall thickening on contrast-enhanced CT is a typical finding in TA (Restrepo et al. 2011). On MRI, thickened walls can be observed, in addition to mural edema (Desai et al. 2005; Nastri et al. 2004).

[F-18]fluorodeoxyglucose ([F-18]FDG) positron emission tomography/computed tomography (PET/CT) imaging can be used to detect inflammatory processes in the vascular wall; this technique can identify more lesions than MRI (Webb et al. 2004; Walter et al. 2005; Meller et al. 2003). When biopsy, US, and MRI findings are unremarkable, additional examination with [F-18]FDG PET/CT can reveal TA-related inflammation. Reports have shown that [F-18]FDG PET/CT is useful for the detection of active inflammation in patients with TA and can be used to assess the response to steroid treatment (Tezuka et al. 2012; Kobayashi et al. 2005). As TA is a pan-arteritis disorder, [F-18]FDG PET/CT could be appropriate for assessing whole-body inflammatory processes. Approximately 30% of patients with TA have other chronic inflammatory diseases, such as Hashimoto disease or inflammatory bowel disease (Kerr et al. 1994). In patients with giant cell arteritis, splenic [F-18]FDG uptake is elevated, and this can be treated using steroids (De Winter et al. 2000). The use of [F-18]FDG PET/CT in the diagnosis of TA has been increasing; therefore, it is essential to understand extra-vessel findings on [F-18]FDG PET/CT in patients with TA. To the best of our knowledge, there have been no studies regarding extra-vessel findings on [F-18]FDG PET/CT in TA patients. Hence, this study aimed to describe the extent and distribution of extra-vascular [F-18]FDG uptake on [F-18]FDG PET/CT in TA patients.

## Methods

### Ethics

This study protocol was approved by the institutional ethics review committee of Tokyo Medical and Dental University, and the requirement for written informed consent was waived.

### Study population

This was a retrospective study. Sixty-four consecutive patients with a total of 73 [F-18]FDG PET/CT scans obtained at our university hospital from 2006 to 2016 (59 women, five men; mean age, 35.4 years; range, 13 to 71 years) were included in the study. A diagnosis of TA was made using the American College of Rheumatology criteria (Arend et al. 1990) and the Guideline for Management of Vasculitis Syndromes (Japanese Circulation Society 2011), as described in a previous study (Tezuka et al. 2012). Disease activity was defined based on the National Institutes of Health criteria (Kerr et al. 1994). Forty age-matched individuals without TA (36 women, four men; mean age, 37.8 years; range, 13 to 70 years) were recruited as the control group, and one [F-18]FDG PET/CT scan from each individual was examined.

### Imaging protocol

Patients fasted for at least 4 h before the examination and were confirmed to have glucose level < 200 mg/dl before the administration of [F-18]FDG. A total of 3.7 MBq/kg of [F-18]FDG was administered intravenously 1 h before the scan. Whole-body images were acquired using Aquiduo (Toshiba Medical, Tokyo, Japan), consisting of a combination of a full-ring PET scanner with lutetium oxyorthosilicate crystals and a 16-row multiple-detector CT scanner. The CT parameters for attenuation correction were as follows: tube voltage of 120 kV, tube current of 150 mA, field of view of 500 mm, pitch of 15.0, and slice thickness of 2.0 mm. PET emission data were obtained in the 3D mode using the following parameters: 2 min in each bed position (for 16 to 18 min in total), matrix size of 256 × 256, and Gaussian filter size of 5 mm.

### Image interpretation

Two board-certified nuclear medicine physicians examined the PET images. We graded [F-18]FDG uptake in large vessels using a four-point scale, as described in a previous study, with accumulation in the liver as a reference (Walter et al. 2005) (Slart 2018). The grades were as follows: 0, no uptake present; 1, low-grade uptake (uptake present, but lower than that in the liver); 2, intermediate-grade uptake (uptake similar to that in the liver); and 3, high-grade uptake (uptake higher than that in the liver). We defined extensive inflammation as involvement of the aorta and major aortic branches. We defined limited inflammation as either involvement of the aorta or involvement of major aortic branches.

We evaluated extra-vessel findings using uptake in the mediastinum blood pool as a reference, except for the spleen. [F-18]FDG accumulation higher than that in the mediastinum blood pool was considered significant. To diagnose splenic involvement, we considered splenic uptake greater than hepatic uptake to be positive. FDG uptake was classified into 2 patterns: focal and diffuse.

We evaluated the relationship between disease activity according to National Institutes of Health score with extra-vessel findings, as well as other inflammatory markers (e.g., white blood cell count (WBC) count, CRP, and ESR).

### Statistics

Categorical variables are denoted as frequencies or percentages, while continuous variables are presented as means ± standard deviation. Disease activity, extra-vessel findings, and other inflammatory markers were compared using Fisher’s exact test for categorical variables and the independent-samples *t* test and Mann–Whitney *U* test for continuous variables. A *P* value less than 0.05 was considered to be statistically significant.

## Results

### Study population

Clinical characteristics of the study population are described in Table 1. Fifty-five of 73 scans showed indications of active disease (75.3%), while 19 showed the inactive form of the disease (24.7%). Forty-four of 73 scans (60.2%) were obtained from patients undergoing steroid treatment, and 29 (59.7%) were from patients not undergoing steroid treatment.

**Table 1:** Baseline characteristics of patients (64 patients, 73 scans)

Mean age (years)	35.4 (13–71)
Sex	
Male	5 (7.8%)
Female	59 (92.2%)
CRP (ng/mL)	1.73 (0–11.4)
ESR (mm/hour)	40.3 (2–134)
WBC (μL)	9133 (4200–16,600)
Hemoglobin(g/dl)	11.9 (6.9–15.6)
Disease activity	
Active	54 (75.3%)
Inactive	19 (24.7%)
Steroid treatment	44 (60.2%)
Immunosuppressive therapy	15 (20.5%)
Ulcerative colitis	5 (6.8%)

*CRP* C-reactive protein, *ESR* erythrocyte sedimentation rate. Values in parentheses show range or percentage

### Whole-body [F-18]FDG PET/CT findings

Vascular involvement was present in 55 of 73 scans (75.3%). Among these, 18 (24.7%) showed Grade 1, 24 (32.9%) showed Grade 2, and 13 (17.8%) showed Grade 3 (Table 2). [F-18]FDG uptake in vessels was detected in a median of 2.1 (range, 0 to 8) monitored sites. Twenty-seven of 73 scans (37.5%) showed extensive inflammation, with involvement of the aorta and major aortic branches. In contrast, 28 of 73 (38.3%) scans revealed limited inflammation (either involvement of the aorta or involvement of major aortic branches).

**Table 2:** Vascular involvement on PET/CT scan (*n* = 73)

Artery uptake^※^	
Overall	55 (75.3%)
Grade 0	18 (24.7%)
Grade 1	18 (24.7%)
Grade 2	24 (32.9%)
Grade 3	13 (17.8%)
Vascular (V), Extra-vascular (E) pattern	
V+E	8
V	13 (18.1%)
E	8 (11.1%)
None	10 (13.9%)

*PET/CT* positron emission tomography/computed tomography

^※^Maximum grade values of vascular involvement in each patient

**Fig. 1:**
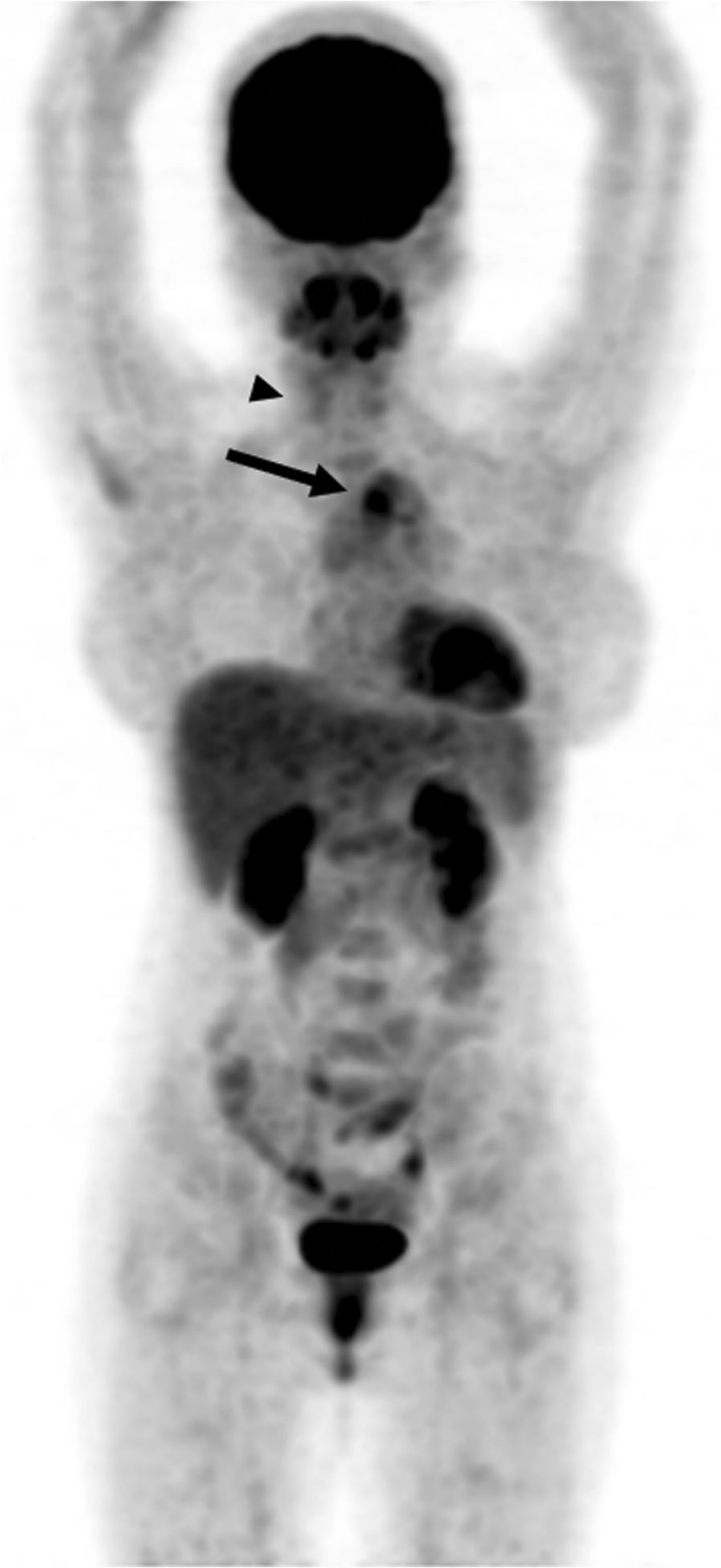
[F-18]FDG PET/CT scan of a 22-year-old woman diagnosed with Takayasu arteritis. [F-18]FDG uptake is present in the aortic arch (arrow). Note the diffuse [F-18]FDG uptake in thyroid glands, indicative of thyroiditis (arrowheads)

**Fig. 2:**
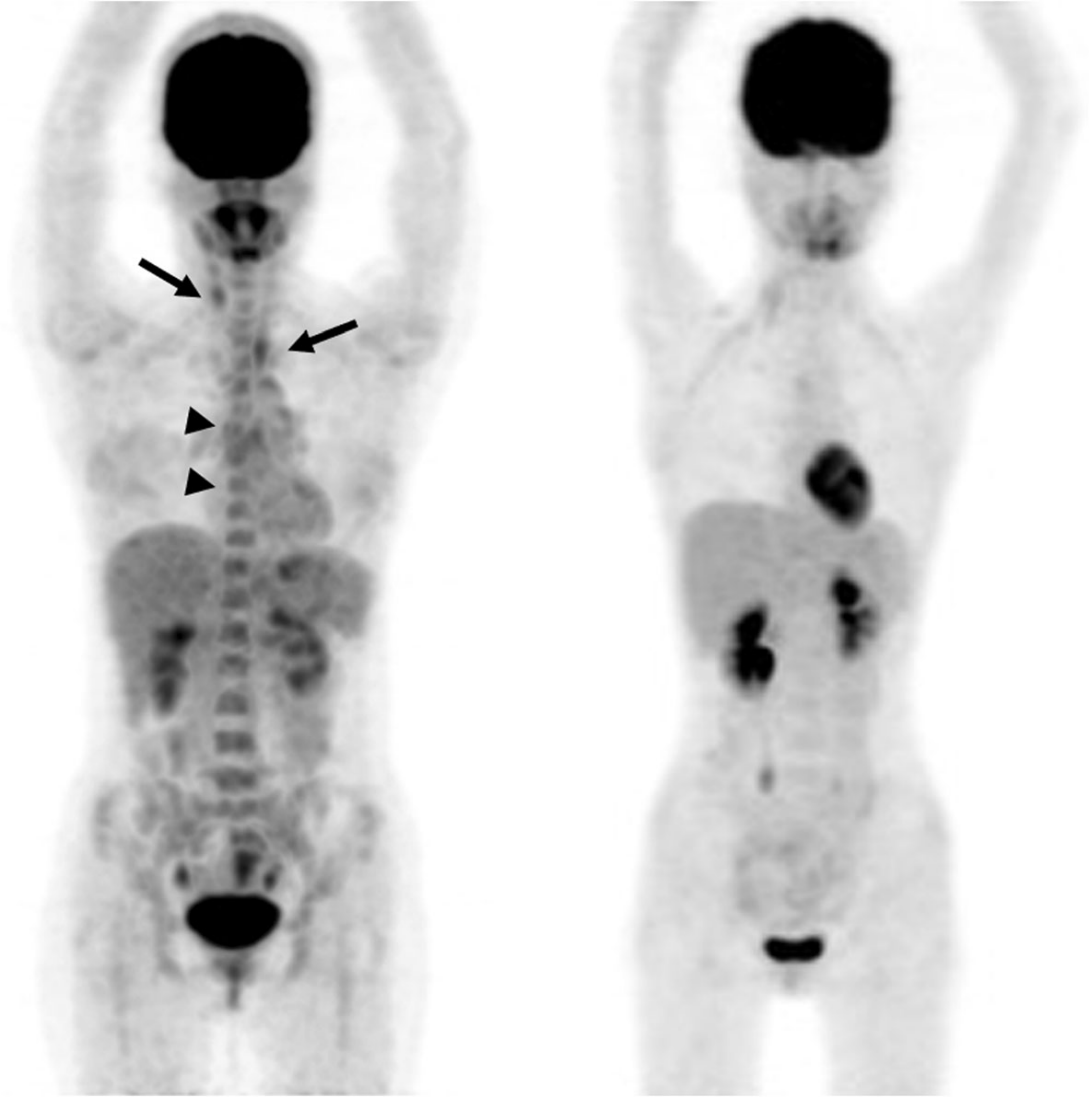
[F-18]FDG PET/CT scan of an 18-year-old woman diagnosed with Takayasu arteritis and an age-matched control patient. [F-18]FDG uptake is present in the right and left common carotid arteries and aortic arch (arrows). Note the [F-18]FDG uptake in vertebrae and pelvic bones (arrowheads), compared with the control

Extra-vessel involvement was present in 50 of 73 (68.4%) scans, specifically at the following sites: lymph nodes, 1/73 (1.4%); thyroid glands, 13/73 (17.8%); thymus, 8/73 (11.0%, Fig. 1); spleen, 1/73 (1.4%); and bone marrow, 33/73 (55.6%). Among patients who showed FDG uptake in thyroid glands, one patient who demonstrated diffuse FDG uptake in bilateral thyroid gland was diagnosed with papillary carcinoma (1/13, 7.7%), and 4 patients had abnormal thyroid function (3/13, 23.1%). Bone marrow involvement (Fig. 2) was present in 40 (54.8%) scans: vertebrae alone in 33 (45.2%) and both vertebrae and pelvic bones in 7 scans (9.6%). All findings are summarized in Table 3. No tumors or other diseases were identified in bone marrow during follow-up. In addition, [F-18]FDG uptake was present in the pharynx, breast tissue, ovary, and uterus; however, this was considered to be physiological uptake.

**Table 3:** Extra-vessel FDG uptake on ^18^F FDG PET/CT scans (*n* = 73)

Thyroid glands	Overall	13 (17.8%)
	Focal	4 (5.5%)
	Diffuse	9 (12.3%)
Bone marrow	Overall	40 (54.8%)
	Vertebrae alone	33 (45.2%)
	Vertebrae + pelvic bones	7 (9.6%)
	Pelvic bones alone	0 (0%)
Thymus		8 (11.0%)
Spleen		1 (1.4%)
Lymph nodes		1 (1.4%)

*FDG* fluorodeoxyglucose, *PET/CT* positron emission tomography/computed tomography

Vessel and extra-vessel involvement were present both individually and in combinations: combined vessel and extra-vessel involvement was detected in 41/73 (56.9%) scans, vessel involvement alone in 13/73 (18.1%) scans, and extra-vessel involvement alone in 8/73 (11.1%) scans. The remaining 10/73 (13.9%) scans showed no involvement.

### Comparison with normal control

Patients with TA had higher [F-18]FDG uptake in the spine (*P* = 0.03) and thyroid glands (*P* = 0.003) than did the normal controls. [F-18]FDG uptake in pelvic bones (*P* = 0.425), thymus (*P* = 1), spleen (*P* = 0.5), and lymph nodes (P = 1) was comparable between groups (Table 4).

**Table 4:** Comparison of extra-vessel FDG uptake between patients with Takayasu arteritis and controls

	TA (*n* = 40)	Control (*n* = 40)	*p* value
Spine	19	10	0.034
Pelvic bone	4	2	0.425
Thyroid glands	10	1	0.003
Thymus	2	2	1.000
Spleen	1	0	0.500
Lymph nodes	1	1	1.000

*FDG* fluorodeoxyglucose

### Disease activity

Patients with active TA had a higher number of [F-18]FDG uptake sites in the vasculature than did those with inactive TA (*P* = 0.001) (Table 5). The number of extra-vessel findings was not significantly correlated with disease activity (*P* = 0.542). Although the existence of extra-vessel involvement was not correlated with disease activity, patients with a National Institutes of Health score of ≥ 1 had increased extra-vascular involvement; this difference was statistically significant (*P* = 0.008). Among other inflammatory indicators, CRP level and ESR count were significantly correlated with disease activity (*P* < 0.0001 and *P* = 0.015). WBC count was not significantly correlated with disease activity (*P* = 0.424).

**Table 5:** Factors correlated with disease activity (NIH score ≥ 2)

Number of vascular involvement sites	*P* = 0.001
Number of extra-vascular involvement sites	*P* = 0.542
Extra-vascular involvement	
NIH score ≥ 2	*P* = 0.309
NIH score ≥ 1	*P* = 0.008
CRP	*P* < 0.0001
ESR	*P* = 0.015
WBC	*P* = 0.424

*NIH* National Institutes of Health, *CRP* C-reactive protein, *ESR* erythrocyte sedimentation rate, *WBC* white blood cell count

## Discussion

To our knowledge, the current study is the first report on extra-vessel findings of Takayasu arteritis on [F-18]FDG PET/CT. We identified [F-18]FDG uptake in several extra-vascular regions and showed that TA patients had a greater number of extra-vessel findings than normal controls. We also demonstrated that extra-vessel findings were possibly correlated with disease activity, suggesting that extra-vessel [F-18]FDG uptake may be present in the context of active inflammation in TA patients.

Many patients in the present study showed [F-18]FDG accumulation in the bone marrow of vertebrae and pelvic bones. Elevated [F-18]FDG accumulation in bone marrow is related to bone marrow activation, as well as the elevation of WBC count and CRP level (Inoue et al. 2009). [F-18]FDG accumulation in the bone marrow could also be due to red marrow hyperplasia caused by bleeding anemia or hematopoietic stimulation treatment (Gordon et al. 1997). In the current study, most patients had slightly low hemoglobin count (Table 1), but the extent was not severe. Moreover, TA patients showed greater FDG accumulation in the bone marrow than age-matched controls, indicating that a low hemoglobin level is not the primary factor for FDG uptake in the bone marrow. The inflammatory process caused by TA may affect hyperactivation of the bone marrow in TA patients.

In addition to bone marrow, [F-18]FDG uptake in thyroid glands was observed in the current study. Several studies have reported incidental [F-18]FDG accumulation in the thyroid glands on PET/CT. The frequencies of [F-18]FDG uptake in the thyroid gland were 1.6% for focal uptake and 2.1% for diffuse uptake (Soelberg and Bonnema 2012). Focal uptake was also related to the presence of malignant tumors (Soelberg and Bonnema 2012; King et al. 2007; Chen et al. 2009). In the current study, 17.8% of patients showed [F-18]FDG uptake in the thyroid glands, which is higher than the previously reported frequency in normal individuals. One patient diagnosed with papillary carcinoma also showed FDG uptake in the contralateral thyroid gland. These findings indicated that some factors in TA patients caused increased [F-18]FDG uptake in the thyroid glands. There are few reports on the relationship between TA and thyroiditis. The HLA types A24 and B52 may constitute genetic factors contributing to the coexistence of TA and Hashimoto thyroiditis (Horai et al. 2011).

Eight of 73 scans showed [F-18]FDG accumulation in the thymus. [F-18]FDG uptake in the thymus on PET/CT can be observed in normal patients; thus, increased [F-18]FDG uptake in the thymus can be difficult to interpret (Brink et al. 2001), and morphologic features are useful for differentiating physiological and hyperplastic uptake from malignant uptake. In this study, plain CT did not reveal any lesions in the thymus, suggesting that the uptake might have been physiological or hyperplastic. To the best of our knowledge, a relationship between TA and thymic lesions has not yet been reported.

One patient showed [F-18]FDG uptake in the spleen. Diffuse FDG uptake in the spleen can be observed in inflammatory conditions including infection, bacteremia, and sepsis (Love et al. 2005). Presumably, increased glucose usage by this organ is related to increased splenic activity in the context of infection. Moreover, inflammation caused by TA, or severe anemia (6.9 mg/dl) in the patient, could have caused diffuse FDG uptake in the spleen (Kim et al. 2014).

Only one patient showed [F-18]FDG uptake in the lymph nodes; thus, it is difficult to determine whether TA itself causes such accumulation. In one study, lymph node swelling and hepatomegaly in lymph nodes were present in the pre-pulseless stage of TA (Yotsuyanagi et al. 1995). Lymph node swelling and hepatomegaly are typically related to viral infection, rather than vasculitis, and there is no valid rationale for suspecting that TA causes such symptoms. We believe that [F-18]FDG uptake in the lymph nodes is uncommon in TA patients.

Extra-vessel [F-18]FDG uptake was present in patients with active and non-active forms of TA. The extent of extra-vessel [F-18]FDG uptake was not significantly different between groups. Patients with a National Institutes of Health score of ≥ 1 had a higher number of extra-vessel findings than did patients with a score of zero. Vascular uptake and overall uptake were related to disease activity. Extra-vessel [F-18]FDG accumulation could thus be correlated with disease activity. In some cases, extra-vessel [F-18]FDG uptake reduced or completely resolved after treatment; however, some of these did not respond to treatment. It was previously reported that coexistent inflammatory bowel disease was independent of TA but that erythema nodosum could be resolved after treatment for TA (Kerr et al. 1994). Therefore, extra-vascular findings that do not respond to treatment can still be related to TA. TA patients had greater extra-vessel [F-18]FDG accumulation than normal controls, suggesting that extra-vascular [F-18]FDG accumulation is related to TA-related inflammatory processes. Considering that serological parameters such as ESR and CRP are not specific inflammatory markers of TA activity, extra-vessel findings may be a potential indicator of inflammation in TA.

This study had some limitations. First, the study design was retrospective. Second, many patients were under steroid and immunosuppressive therapy; therefore, disease onset and the highly active phase of TA could not be evaluated. Thirdly, we utilized 4 h of fasting time for all patients. Relatively short fasting time may lead to inadequate suppression of FDG uptake in the mediastinum blood pool and liver. Longer fasting time may render a more precise evaluation of FDG uptake with suppressed and stable FDG uptake in the reference organs. Further research is necessary to understand the relationship between disease activity and extra-vessel [F-18]FDG uptake in TA patients; the clinical applicability of extra-vessel findings should also be evaluated.

## Conclusions

In this study, we characterized extra-vascular [F-18]FDG uptake on [F-18]FDG PET/CT in TA patients. Our results indicated that extra-vessel [F-18]FDG uptake may be present in the context of TA-related inflammatory processes. Information regarding extra-vascular [F-18]FDG uptake may be useful for detection and evaluation of inflammatory processes when interpreting PET/CT scans obtained from TA patients. These findings may increase our understanding of the mechanism of inflammation in TA and the cause of symptoms in affected patients.

## Data Availability

The data are not available for public access because of patient privacy concerns but are available from the corresponding author on reasonable request.

## Abbreviations

CRP: C-reactive protein
ESR: Erythrocyte sedimentation rate
FDG: Fluorodeoxyglucose
PET/CT: Positron emission tomography/computed tomography
TA: Takayasu arteritis
WBC: White blood cell count
